# Pubertal Triptorelin Exposure Alters Hypothalamic Reproductive–Metabolic Integration in a Sex-Specific Manner

**DOI:** 10.3390/biology15161376

**Published:** 2026-08-12

**Authors:** Dunja Dimitrijevic, Ana Milosevic, Nina Bogicevic, Katarina Tesovic, Zeljko Pavkovic, Marija Janjic, Danijela Savic, Ivana Bjelobaba

**Affiliations:** Institute for Biological Research “Siniša Stanković”—National Institute of Republic of Serbia, University of Belgrade, 11108 Belgrade, Serbia; dunja.dimitrijevic@ibiss.bg.ac.rs (D.D.); ana.milosevic@ibiss.bg.ac.rs (A.M.); nina.bogicevic@ibiss.bg.ac.rs (N.B.); katarina.tesovic@ibiss.bg.ac.rs (K.T.); zeljko.pavkovic@ibiss.bg.ac.rs (Z.P.); marija.janjic@ibiss.bg.ac.rs (M.J.); danisto@ibiss.bg.ac.rs (D.S.)

**Keywords:** gonadotropin-releasing hormone, puberty blockers, triptorelin, gender-affirming care

## Abstract

Gonadotropin-releasing hormone agonists are used to treat central precocious puberty and are increasingly prescribed in gender-affirming care. Despite well-established reproductive effects, their broader physiological effects during puberty remain poorly understood. Male and female Wistar rats received a depot triptorelin injection at pubertal onset. Body mass, food intake, and locomotor activity were monitored. After 28 days, reproductive function was assessed, and pituitary and hypothalamic gene expression was analyzed. Changes in gonadal weight, estrous cycle, folliculogenesis, seminiferous tubules’ morphology, and sperm count confirmed the suppression of pubertal maturation. Triptorelin downregulated *Gnrhr* and *Lhb*, demonstrating suppression of gonadotrope function. Despite comparable reproductive suppression, metabolic responses to triptorelin were strongly sex-dependent. Females exhibited increased body weight gain accompanied by delayed hyperphagia, whereas males showed reduced body weight gain, unchanged food intake, and increased mean locomotor speed. Female-specific transcriptional remodeling was observed in the hypothalamus, characterized by downregulation of *Npy* and *Lepr* and upregulation of *Sst* and *Kiss1* expression. Collectively, these findings demonstrate that pubertal triptorelin treatment induces profound reproductive suppression in both sexes while eliciting sex-specific hypothalamic adaptations in pathways regulating energy homeostasis. This suggests that puberty blockers exert broader neuroendocrine effects beyond the suppression of reproductive maturation.

## Simple Summary

Reproduction is primarily regulated by the hypothalamic–pituitary–gonadal axis: gonadotropin-releasing hormone (GnRH), produced by neurons in the hypothalamus, acts on the pituitary gland that produces hormones controlling gonadal (ovarian and testicular) functions. Puberty is the process of reproductive maturation that involves the activation of GnRH neurons and the pulsatile release of GnRH. GnRH agonists (GnRHa) are synthetic molecules similar to GnRH that are used as puberty blockers to treat premature puberty and as a part of treatment in some transgender adolescents. We used GnRHa to treat male and female rats at the start of puberty and examined its effects on reproductive functions and metabolism. GnRHa treatment induced the suppression of reproductive function in rats of both sexes, evidenced by changes in ovaries and testes and in pituitary markers important for the control of reproduction. The effects on metabolism were sex-specific: treated females gained more weight and had a higher food intake than controls, while treated males exhibited decreased weight gain and changes in locomotion compared to controls. Also, only females showed changes in the hypothalamic molecules regulating the energy balance. These results demonstrate that puberty blockers exert broader impact than suppressing reproductive maturation and highlight the importance of further research of their effects.

## 1. Introduction

Puberty is a developmental period characterized by somatic and sexual development of secondary sexual characteristics and reproductive maturation. This process involves the establishment of hypothalamic–pituitary–gonadal (HPG) axis activity and is triggered by the hypothalamic peptide kisspeptin, which plays a paramount role in regulating the pulsatile release pattern of gonadotropin-releasing hormone (GnRH). GnRH acts on its receptor (GnRHR) on the pituitary gonadotropes, which is critical for the synthesis and release of luteinizing (LH) and follicle-stimulating (FSH) hormone. LH and FSH act on the gonads, driving steroidogenesis, as well as folliculogenesis and spermatogenesis [1,2].

The timing of puberty is directly linked to energy stores and nutritional status. Conditions such as extreme exercise and malnutrition can delay puberty, while obesity can lead to an earlier puberty onset [3]. The integration of peripheral and central metabolic signals that govern pubertal maturation occurs within the hypothalamus. The postnatal maturation of hypothalamic neuropeptide Y (NPY)/agouti-related peptide (AgRP) and proopiomelanocortin (POMC) circuits establishes the metabolic framework that enables the central assessment of energy sufficiency, a prerequisite for pubertal activation of the HPG axis. As these circuits reach functional maturity, they integrate signals like leptin and insulin-like growth factor 1 (IGF-1) to trigger kisspeptin neurons, effectively lifting the metabolic brake on the reproductive system and initiating the transition into puberty [3]. Moreover, neuropeptides synthesized by POMC, AgRP and NPY neurons continue to participate in the regulation of reproduction beyond puberty, since these peptides have direct effects on GnRH excitability [4].

Since the seminal work of Knobil in monkeys in 1978 [5], it has been well established that intermittent GnRH release is essential for proper function of the HPG axis. In all mammals investigated so far, continuous application of GnRH leads to pituitary desensitization and suppression of HPG axis function. Specifically, prolonged occupancy of GnRHR induces a striking decrease in LH and FSH secretion, as well as downregulation of mRNA expression of gonadotropin beta subunits [6].

GnRH analogs are synthetic peptides that structurally resemble natural GnRH, with some amino acid modifications. They were developed in the early 1980s and today have a variety of clinical applications, including treatment of prostate cancer and various gynecological conditions [7]. Agonist analogs of GnRH (GnRHa) have increased affinity for GnRHR and a longer half-life compared to the natural peptide. After an initial flare-up in gonadotropin release, GnRHa induce long-term suppression of pituitary gonadotrope function [8].

Since the early 1990s, GnRHa have been the treatment of choice for central precocious puberty (CPP). Commonly referred to as puberty blockers, they delay the onset of secondary sexual characteristics and prevent premature activation of the HPG axis. Another benefit of GnRHa is that they improve final height in girls with CPP [9]. For this reason, they are also used, usually in combination with other agents, in pediatric conditions associated with compromised adult stature, including growth hormone (GH) deficiency, idiopathic short stature, and children born small for gestational age [10]. More recently, the use of GnRHa was extended to transgender adolescents, with the aim of suppressing the development of secondary sexual characteristics associated with biological sex and thus improving gender dysphoria. In addition to this benefit, GnRHa are often viewed as agents that prolong the time available for decisions about gender transition [11].

Although these treatments were long considered safe, their use in minors has become increasingly debated. Several countries, including the UK, Sweden, and New Zealand, have introduced restrictions on the use of puberty blockers in children under 18 years of age. In contrast, in countries such as Belgium and the Netherlands, GnRHa remain part of the established clinical protocol (the Dutch protocol) for the treatment of transgender adolescents. These differing approaches reflect ongoing ethical and medical concerns, particularly regarding the limited understanding of the short- and long-term effects of GnRHa on health and development [12]. Furthermore, data on male subjects remain scarce, as CPP is more frequently diagnosed in girls [13].

In parallel with ongoing clinical investigations [14], preclinical research has increasingly focused on elucidating the effects of GnRHa exposure. Most rodent studies to date have primarily examined the status and reversibility of HPG axis function, often following prepubertal administration [15,16,17,18,19]. However, less attention has been given to GnRHa exposure during puberty itself—a clinically relevant scenario in which ongoing pubertal development is interrupted rather than delayed.

Here, we investigate the effects of pubertal administration of the GnRHa triptorelin on the HPG axis and its integration with metabolic regulatory pathways in rats of both sexes. We demonstrate that prolonged triptorelin exposure suppresses HPG axis activity in both males and females, as evidenced by reduced gonadal weight, altered gonadal histological features, and changes in gonadotrope-related gene expression. Notably, these effects are accompanied by sex-specific metabolic outcomes, including increased body weight and food intake in females but not males. Furthermore, hypothalamic expression of key metabolic regulators, including *Npy* and *Lepr*, is selectively downregulated in females. Collectively, these findings indicate that pubertal triptorelin exposure disrupts hypothalamic reproductive–metabolic integration in a sex-specific manner, highlighting a previously underappreciated dimension of GnRHa action during adolescence.

## 2. Materials and Methods

### 2.1. Animals

The study was conducted on 5–6-week-old female and male rats of the Wistar strain, provided by the Experimental Animals Breeding Facility of the Institute for Biological Research “Sinisa Stankovic”. During the experiment, the animals were housed three per cage and kept in a standard laboratory environment (12 h light/dark cycle, at constant temperature and humidity), with unlimited access to chow and tap water. The experimental protocols were approved by the National Licensing Committee at the Department of Animal Welfare, Veterinary Directorate, Ministry of Agriculture, Forestry and Water Management of the Republic of Serbia (Permit No 323-07-11135/2022-05) and performed in compliance with the guidelines of the Directive on the Protection of Animals Used for Experimental and Other Scientific Purposes (2010/63/EU). The experimental procedures did not result in any unexpected adverse events or signs of pain or distress. Animals were monitored daily using non-invasive procedures, including assessment of puberty onset, body weight, and estrous cycle. The first experiment for estrous cycle assessment, gonadal morphology, body weight gain and quantitative real-time PCR (q-PCR) analyses was performed with 7 animals per group. The second experiment for food intake and locomotor activity was done with 9 animals per group. In total, 64 animals were used (32 females and 32 males).

### 2.2. Triptorelin Administration

In female rats, puberty onset typically occurs between postnatal days (PND) 30 and 40 and is determined by vaginal opening. In male rats, puberty onset occurs between postnatal days 38 and 45 and is determined by preputial separation, i.e., the separation of the prepuce from the glans penis [1]. Vaginal opening was registered between PND 30 and 36 (33.5 ± 2.2), while preputial separation occurred between PND 39 and 45 (42.0 ± 2.2). On the day of puberty onset, animals were randomly assigned to the Control (C) or Triptorelin (T) group. Animals in the T group received an intramuscular injection of 100 μL of Diphereline^®^ (Ipsen Pharma Biotech, Paris, France; active substance: triptorelin pamoate) dissolved in sterile saline solution at a dose of 2.9 mg/kg body weight [20]. Animals in the C group received 100 μL of vehicle, i.e., sterile saline solution i.m. at the onset of puberty.

### 2.3. Estrous Cycle Assessment

Starting from the day of vaginal opening, non-invasive vaginal lavage was performed daily between 9:00 and 10:00 a.m. The phase of the estrous cycle was determined based on the cellular composition and morphology, which was assessed via light microscopy according to Shreya et al. [21]. All females were sacrificed during the diestrus phase of the estrous cycle.

### 2.4. Food Intake Determination

For the calculation of food intake, animals were housed 3 per cage according to their assigned experimental group. Food consumption per cage (*n* = 3 cages/group) was calculated as the difference between the amount of chow provided and the amount remaining after 48 h. The data are expressed as food consumption per animal over the 48 h and presented as mean ± SEM. The experimenter was blinded to the treatment group allocation during food intake determination and data analysis.

### 2.5. Novel Rectangular Arena Procedure

Locomotor activity was assessed 26 days after the triptorelin or vehicle injection, using an automated monitoring system consisting of Opto-Varimex 5 cages (Columbus Instruments, Columbus, OH, USA) connected to an IBM-compatible computer for real-time data acquisition. Prior to testing, all animals were acclimated to the experimental room for 30 min to minimize the influence of environmental novelty. Following habituation, each animal was placed in the center of the testing arena and allowed to freely explore the novel environment for 30 min. Behavioral data were collected and analyzed using Auto-Track software (version 5.5.9). Locomotor activity was quantified as the total distance traveled, calculated from consecutive interruptions of infrared beams within the apparatus. Average locomotor speed (cm/s) was automatically calculated by the tracking software as the total distance traveled in centimeters divided by the duration of active ambulatory time over the 30 min test period. The results are presented as mean ± SEM, from *n* ≥ 8 animals per group. The experimenter was blinded to the treatment group allocation during behavioral testing and data analysis.

### 2.6. Tissue Collection

Four weeks after triptorelin administration, the animals were sacrificed by asphyxiation in a CO_2_ chamber with a gradual increase in CO_2_ concentration. The animals were perfused with 50 mL of cold saline. From each animal, the pituitary gland and hypothalamus were isolated, weighed, and stored in RNAlater^®^ (Sigma-Aldrich, Saint Louis, MO, USA) at −80 °C for subsequent RNA isolation and gene expression analysis. The hypothalamus was dissected on ice as a block of tissue, with boundaries defined using a coronal rat brain matrix. The rostral cut was made 2 mm anterior to the optic chiasm, and a caudal cut was made behind the mammillary bodies. Lateral borders were set to 2 mm lateral to the third ventricle. The dorsal border of the sampled block was defined by the top of the third ventricle. Ovaries and testes were collected from all animals, fixed in Bouin’s solution, and subsequently processed for histological analysis.

### 2.7. Spermatozoid Count and Gonadal Histology

Heads and tails of the epididymis were macerated in DMEM medium (Gibco, Thermo Fisher Scientific, Waltham, MA, USA) and incubated at 34 °C for 20 min on a shaker at 700 rpm. The suspension was strained through a 100 µm mesh (Corning, NY, USA), and spermatozoa were counted on a Neubauer hemocytometer according to Kempinas and Lamano-Carvalho [22]. The results are presented as mean numbers of spermatozoa per head (caput) or tail (cauda) of one epididymis ± SEM, from *n* = 7 animals per group.

After fixation, testes and ovaries were embedded in paraffin, cut to 7 µm sections and mounted on gelatin-coated slides. Ovary and testis sections were stained with hematoxylin and eosin (H&E) and examined and photographed using a Zeiss Axiovert microscope (Carl Zeiss GmbH, Vienna, Austria). Every 10th ovarian section was used to analyze ovarian morphology, which was performed according to criteria described by Pedersen and Peters [23]. According to this classification, eight follicular types are distinguished and grouped into small (types 1–3a), medium (types 3b–5a), and large (types 5b–8) follicles. Antral follicles were analyzed because the development of small and early medium follicles is largely gonadotropin-independent, whereas the transition to the antral stage and subsequent growth of antral follicles become increasingly dependent on pituitary gonadotropins [24]. Antral follicles with a cavity within the granulosa cell layer (types 6 and 7), and large corpora lutea (CL) (over 1000 µm in diameter) were counted and expressed as the total numbers per ovary. The results are presented as mean counts ± SEM, from *n* ≥ 6 ovaries per group. Inner and outer diameters of seminiferous tubules and tubule wall thickness were measured from 60 to 80 round or close to round tubules from 7 to 10 micrographs (10× magnification) of H&E-stained sections, per animal [25]. The results are expressed as mean diameters ± SEM, from *n* ≥ 6 testes per group. Micrographs were sized, cropped and arranged in Photoshop CS (Adobe Inc., San Jose, CA, USA). The experimenters were blinded to the treatment group allocation during spermatozoid count and histological analysis.

### 2.8. RNA Isolation, Reverse Transcription and Gene Expression

Total pituitary RNA was isolated using the RNeasy Mini Kit (QIAGEN, Hilden, Germany) according to the manufacturer’s instructions, while hypothalamic RNA was isolated using the TRIzol method. Briefly, 1 mL of TRIzol™ Reagent (Invitrogen, Carlsbad, CA, USA) was added per 100 mg of tissue, followed by tissue homogenization. Chloroform was added at one-fifth the volume of TRIzol, and samples were vortexed and centrifuged (12,000× *g*, 4 °C, 15 min). The aqueous phase containing RNA was carefully transferred to a new tube, mixed with half-volume isopropanol and stored at −80 °C overnight. The following day, samples were thawed and centrifuged (12,000× *g*, 4 °C, 10 min). RNA pellets were washed with 75% ethanol and centrifuged (7500× *g*, 4 °C, 5 min), air-dried, and resuspended in UltraPure water (Milli-Q^®^, MilliporeSigma, Burlington, MA, USA). RNA concentration was determined using a NanoPhotometer MicroVolume spectrophotometer (Implen, Munich, Germany, catalog number: N60 UV/Vis), while sample purity was assessed by A260/A280 and A260/A230 ratios.

Next, reverse transcription was carried out with the High-Capacity cDNA Reverse Transcription Kit (Applied Biosystems by Thermo Fisher Scientific, Waltham, MA, USA) in a total reaction volume of 20 µL, containing 1 µg of RNA. According to the manufacturer’s instructions, the reaction was performed under the following conditions: 10 min at 25 °C; 120 min at 37 °C; 5 min at 85 °C; and a final hold at 4 °C. q-PCR was performed using TaqMan^®^ or SYBR™ Green reagents in 96-well plates (all from Applied Biosystems by Thermo Fisher Scientific, Waltham, MA, USA) on the QuantStudio™ 3 Real-Time PCR System (Applied Biosystems by Thermo Fisher Scientific, Waltham, MA, USA). The reaction contained 10 ng of cDNA in a total volume of 10 µL, and the thermocycler conditions were as follows: 2 min at 50 °C, 10 min at 95 °C, and 40 cycles of 15 s at 95 °C followed by 1 min at 60 °C. For the SYBR™ Green method, after the 40 amplification cycles, a final melt curve stage was performed, consisting of 15 s at 95 °C and 1 min at 60 °C. Information on primers and probes is provided in Table 1 and Table 2, respectively. Amplification efficiency of custom-made primer pairs was determined using serial dilutions of pooled cDNA. All tested primers had efficiency rates between 92% and 102%. Relative gene expression levels were calculated using the comparative 2^−∆Ct^ method, with glyceraldehyde 3-phosphate dehydrogenase (*Gapdh*) as the reference gene, and then log_2_-transformed.

### 2.9. Statistical Analysis

Parametric analyses were performed using R (version 4.4.2, packages: lme4, lmerTest, car, emmeans, pheatmap), while GraphPad Prism 8 was used for Welch’s *t*-tests and data visualization. Model assumptions (normality and homoscedasticity) were verified using residual diagnostics, Shapiro–Wilk, and Levene’s tests. Prior to analysis, extreme biological and technical outliers were identified and excluded. Accordingly, one female with a highly irregular estrous cycle was excluded from all analyses.

Depending on the experimental design, specific statistical models were applied as follows:

For body mass and food intake, linear mixed-effects models were used to account for repeated measurements over time, with Sex, Treatment, Day (categorical factor), and all interactions specified as fixed effects. For body mass, animal identity was included as a random intercept, whereas for food intake (recorded per cage), Cage_ID was specified as a random intercept. Omnibus testing was evaluated via Type III ANOVA with Satterthwaite’s method. Post hoc pairwise comparisons between control and treated groups were conducted within each Sex and Day combination, with *p*-values adjusted using the Šidák correction.

Locomotor activity (total *n* = 35, following exclusion of one outlier) and log_2_-transformed gene expression data were analyzed via Type III two-way ANOVA using sum-to-zero contrasts (contr.sum). Pairwise post hoc differences for all parameters were assessed using Tukey’s adjustment. For gene expression, omnibus *p*-values were adjusted across all genes using the Benjamini–Hochberg (FDR) method and reported as *q*-values. Expression patterns were visualized via hierarchical clustering heatmaps (z-score normalization, Euclidean distance, Ward’s ‘ward.D2’ method).

Data are presented as mean ± SEM. Statistically significant differences are denoted as follows: * *p* < 0.05, ** *p* < 0.01, *** *p* < 0.001.

## 3. Results

### 3.1. The Effect of Triptorelin on Estrous Cycle, Sperm Count and Gonadal Morphology

**Figure 1:**
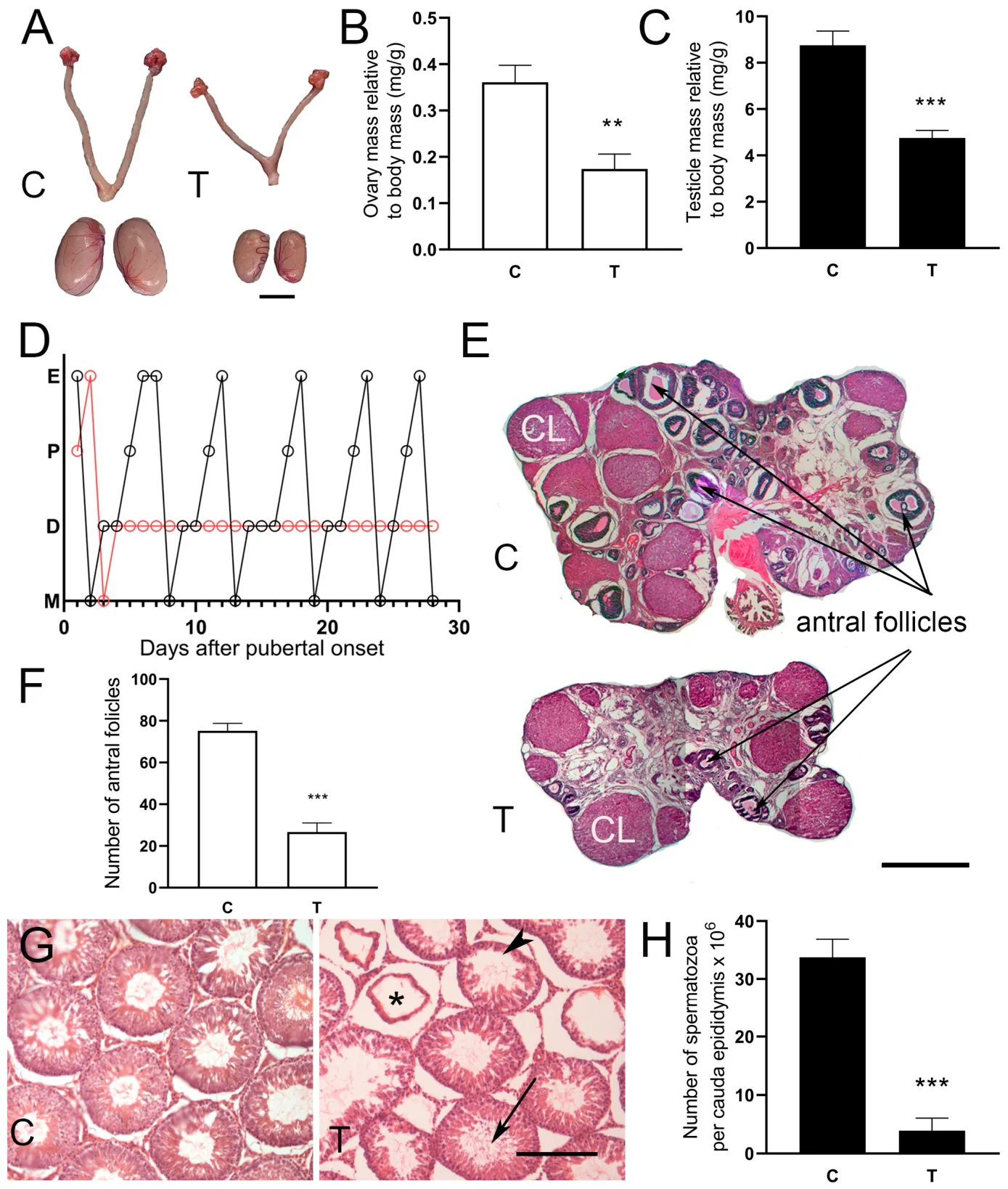
Triptorelin depot-induced changes in gonads in both sexes after 28 days. (**A**, **up**) Representative images of ovaries and uterus in control (C) and triptorelin-treated (T) animals. (**A**, **down**) Representative images of testicles in control and triptorelin-treated males. (**B**) Mass of ovaries relative to body mass in mg/g (mean ± SEM, *n* ≥ 6 /group). (**C**) Mass of testicles relative to body mass in mg/g (mean ± SEM, *n* ≥ 6 /group). (**D**) Representative graphical representation of the estrous cycle in a triptorelin-treated female, arrested in diestrus (red line), and a normally cycling control female (black line) (M—metestrus, D—diestrus, P—proestrus, E—estrus). (**E**) Representative H&E-stained sections of ovaries from the C and T groups. Arrows point to antral follicles; CL-corpus luteum. (**F**) Number of antral follicles per ovary was significantly lower in the T group (mean ± SEM, counted from 70 µm apart slices throughout the whole ovary, from *n* = 6 ovaries/group). (**G**) Representative H&E-stained testicular sections from the C and T groups. Seminiferous tubules in the T group showed various signs of degeneration, such as thinning of the tubule walls (arrowhead) and complete tubule degeneration with the absence of spermatocytes (asterisk). Tubules without signs of degeneration were also observed (arrow). (**H**) The number of spermatozoa per cauda epididymis (mean ± SEM, from *n* ≥ 6 animals/group). ** *p* < 0.01, *** *p* < 0.005; Welch’s *t*-test. Scale bars: 1 cm in (**A**), 1 mm in (**D**), 200 µm in (**H**).

Triptorelin significantly reduced the absolute mass of both ovaries (control 74.9 ± 8.6 mg; triptorelin 40.7 ± 7.3 mg) and testes (control 3.4 ± 0.2 g; triptorelin 1.7 ± 0.1 g). In both males and females, the relative mass of ovaries and testicles in triptorelin-treated animals was significantly decreased compared to control animals (*p* = 0.0041 and *p* = 0.0003, respectively; Figure 1A–C).

From the day of vaginal opening until the end of the experiment, control females maintained regular 4–5-day-long cycles. After triptorelin administration, females completed the ongoing cycle but then remained in diestrus until the end of the experiment. Estrous cycles of representative animals from the C and T groups are shown in Figure 1D. Triptorelin treatment also altered ovarian histology. Representative low-power magnification micrographs depicting H&E-stained ovarian sections from the C and T groups are shown in Figure 1E. Triptorelin-treated animals had a significantly lower number of antral follicles (26.7 ± 4.3) compared to controls (75.2 ± 3.6, *p* < 0.0001; Figure 1F). The antral follicle diameter was also significantly reduced in triptorelin-treated animals (268.8 ± 11.0 µm) compared to controls (380.4 ± 12.1 µm, *p* < 0.005; Figure S1A in the Supplementary Materials File). Atretic follicles and follicular and luteal cysts were frequently observed in triptorelin-treated animals. Ovaries of triptorelin-treated animals were not completely devoid of CL, but their number was significantly lower (12.0 ± 3.9) than in control animals (35.3 ± 1.9, *p* < 0.005). The number of large CL (over 1000 µm in diameter) was also low in triptorelin-treated animals (2.0 ± 0.9) compared to controls (13.7 ± 2.0, *p* < 0.01; Figure S1B). Type 8 follicles were never observed, as all animals were in the diestrus stage. The number of smaller follicles (type 1–5b) was not assessed.

Representative low-power micrographs of H&E-stained testicle sections from the C and T groups are shown in Figure 1G. While some seminiferous tubules were apparently unaffected by treatment (normal structure of the tubule wall and presence of spermatozoa in the lumen (arrow in Figure 1G)), tubules with various signs of degeneration were also observed (arrowhead in Figure 1G). The layered structure of the wall was occasionally completely disrupted, and these tubules were completely devoid of spermatocytes (Figure 1G, asterisk). Degenerative changes were more often observed in the middle portions of the sections. Triptorelin treatment significantly reduced the total diameter of seminiferous tubules (217.2 ± 6.1 vs. 255.7 ± 3.7 µm in controls, *p* < 0.01; Figure S1C), the thickness of the tubule epithelium (52.3 ± 1.7 vs. 69.9 ± 2.5 µm in controls, *p* < 0.005; Figure S1D), and the diameter of seminiferous tubule lumen (91.0 ± 3.1 vs. 114.7 ± 2.8 µm in controls, *p* < 0.005; Figure S1E). The number of spermatozoa in the head and tail of the epididymis was significantly decreased in triptorelin-treated animals (16.4 ± 4.6 × 10^6^ (caput); 3.9 ± 2.1 × 10^6^ (cauda)) compared to controls (48.2 ± 2.6 × 10^6^ (caput); 33.8 ± 3.1 × 10^6^ (cauda), *p* < 0.0001; Figure 1H). In three triptorelin-treated animals, complete absence of spermatozoa was noted in the cauda epididymis.

### 3.2. Gene Expression Profiling of the Pituitary Gland

**Figure 2:**
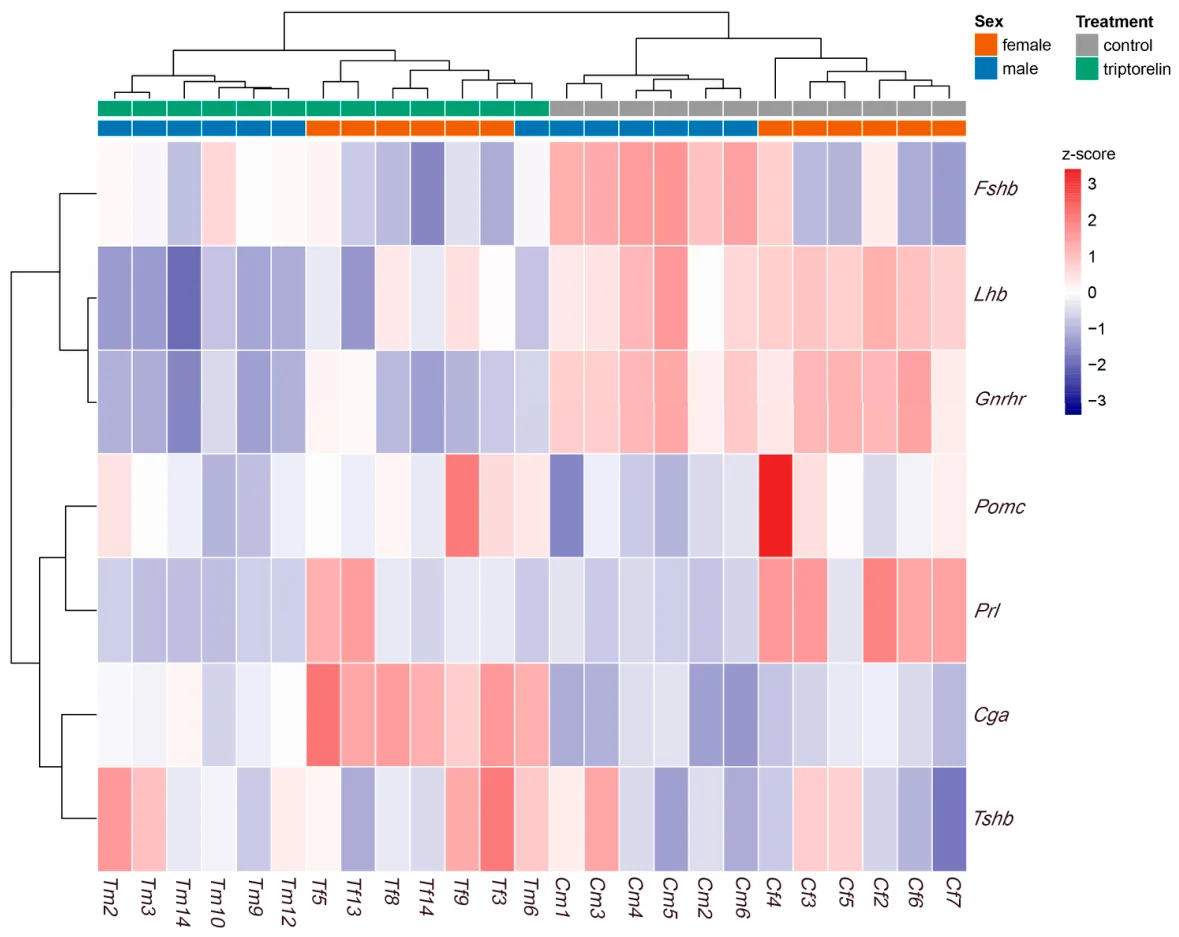
Heatmap of pituitary gene expression. Heatmap showing relative expression levels of selected genes (*Fshb*, *Lhb*, *Gnrhr*, *Pomc*, *Prl*, *Cga*, and *Tshb*) in pituitary samples from control and triptorelin-treated male and female animals. Gene expression values were log_2_-transformed and standardized as z-scores per gene to allow comparison across samples. Rows represent individual genes, while columns represent individual samples (*n* = 25). Samples are annotated by sex (female: orange, male: blue) and treatment (control: gray, triptorelin: green). Hierarchical clustering was performed using Euclidean distance and ward.D2. Color scale: dark blue—downregulation; white—no change; red—upregulation. Clustering patterns suggest that samples group primarily according to treatment, with additional separation by sex. Experimental group abbreviations: Cf —Female Control; Tf—Female Triptorelin; Cm—Male Control; Tm—Male Triptorelin. Numbers denote individual animals within each group.

Having established the impact of triptorelin on gonads, we next examined global gene expression patterns in the pituitary gland. Data were visualized using a heatmap (Figure 2) based on z-score standardized values for each gene. Hierarchical clustering showed that the primary factor driving sample grouping was the treatment effect. This separation was mainly characterized by downregulation of *Lhb* and *Gnrhr*, along with upregulation of *Cga* in treated samples. Within these treatment-defined clusters, a secondary separation by sex was observed, driven by the expression of *Fshb*, *Prl*, and *Pomc*. Overall, these results demonstrate that triptorelin treatment has a more profound effect on the pituitary gene expression profile than biological sex. Hierarchical clustering of genes yielded two primary clades: one comprising *Lhb*, *Fshb*, and *Gnrhr*, reflecting their response to GnRHa-induced suppression, and a second clade containing *Cga*, *Prl*, *Pomc*, and *Tshb*, characterized by more diverse regulatory patterns, including upregulation and sexual dimorphism.

The statistical analysis summary of the effects of sex, treatment and their interaction on the expression profile of selected pituitary genes is presented in Table 3, and post hoc comparisons are presented in Figure S2 in the Supplementary Materials File.

#### 3.2.1. Pituitary Genes Influenced by Both Sex and Treatment

A key subset of pituitary genes, specifically those encoding the gonadotropin subunits—*Lhb*, *Fshb*, and *Cga*, as well as the lactogenic hormone *Prl*—demonstrated significant sensitivity to both biological sex and triptorelin treatment (*q* < 0.05, Table 3).

For *Lhb* and *Cga*, treatment was the primary driver of changes in expression (F_1,21_ = 55.41, *q* < 0.001 and F_1,21_ = 70.21, *q* < 0.001, respectively, Table 3). Although significant sexual dimorphism was present for both genes (*q* = 0.009 for *Lhb* and *q* < 0.001 for *Cga*), the absence of a significant sex-by-treatment interaction *(q* > 0.05) indicates that triptorelin induced a consistent, yet divergent, response in males and females: a robust downregulation of *Lhb* and a significant upregulation of *Cga*, confirmed by post hoc analysis (Table 3, Figure S2). While there were no baseline differences between control groups for *Lhb* (*p* = 0.870) or *Cga* (*p* = 0.460), the treated groups showed a clear divergence, with triptorelin-treated females exhibiting significantly higher expression levels than their male counterparts (*p* = 0.007 and *p* < 0.001, respectively; Figure S2).

For *Fshb* and *Prl*, biological sex accounted for the largest proportion of total variance (F_1,21_ = 32.61, *q* < 0.001 and F_1,21_ = 33.20, *q* < 0.001, respectively; Table 3), reflected in strong dimorphism between control females and males (*p* < 0.001 for both, Figure S2). Triptorelin treatment significantly modulated the expression of both *Fshb* (*q* = 0.003) and *Prl* (*q* = 0.033; Table 3). Post hoc comparisons showed that triptorelin significantly reduced *Fshb* expression in males (*p* = 0.001), whereas its impact on *Prl* downregulation was significantly pronounced in females (*p* = 0.033; Figure S2).

#### 3.2.2. Pituitary Genes with Single-Factor or No Significant Effects

In contrast to the multi-factor responsiveness of the gonadotropin subunits, the expression of *Gnrhr*, *Pomc*, and *Tshb* showed more restricted regulatory patterns.

The expression of *Gnrhr* was modulated only by the treatment effect (F_1,21_ = 85.49, *q* < 0.001). Post hoc analysis confirmed downregulation of *Gnrhr* in triptorelin-treated rats of both sexes compared with their respective controls (*p* < 0.001, Figure S2), while there were no significant differences between sexes, either under control conditions or after treatment (Table 3, Figure S2).

Conversely, the expression of *Pomc* was influenced solely by biological sex (F_1,21_ = 6.93, *q* = 0.022), although post hoc analysis did not reveal significant differences between female and male groups. Finally, *Tshb* expression remained stable—no significant effects were observed for sex, treatment, or their interaction (Table 3). Post hoc comparisons found no significant differences between control and treated groups of either sex.

### 3.3. Body Mass Changes Under Triptorelin in a Sex-Specific Manner

**Figure 3:**
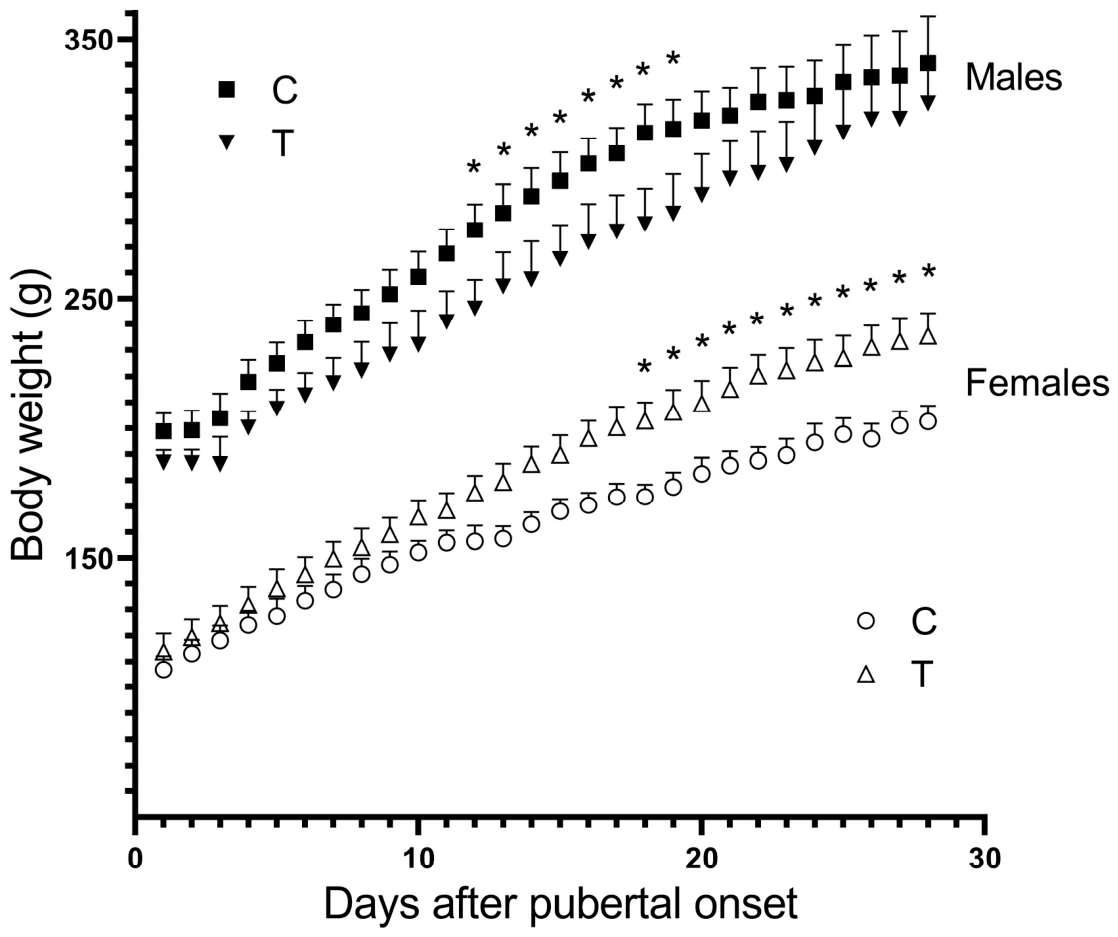
Sex-specific effects of triptorelin on body weight over time. Body weight of male and female rats treated with triptorelin or vehicle was monitored over 28 days. A linear mixed model revealed a significant sex × treatment × day interaction. Post hoc analysis (Šidák correction) showed that triptorelin significantly reduced body weight gain in males between days 12–19 and increased body mass in females between days 18–28 (* *p* < 0.05 vs. control at corresponding time points). Data are presented as mean ± SEM (*n* = 7 per group).

After characterizing pituitary transcriptional responses, we next examined how triptorelin affects the organismal level, beginning with longitudinal tracking of body mass and food intake. Body mass was analyzed using a linear mixed model with sex, treatment, and day as fixed effects and animal identity as a random effect (Figure 3). A significant main effect of sex (F_1,24_ = 111.21, *p* < 0.0001) and day (F_27,648_ = 449.74, *p* < 0.0001) was observed, whereas the main effect of treatment was not significant (F_1,24_ = 0.02, *p* = 0.888).

Significant interactions were detected between sex and treatment (F_1,24_ = 5.83, *p* = 0.024), sex and day (F_27,648_ = 11.55, *p* < 0.0001), and treatment and day (F_27,648_ = 1.80, *p* = 0.008). Importantly, a significant three-way interaction between sex, treatment, and day was observed (F_27,648_ = 3.30, *p* < 0.0001).

According to the post hoc test (Šidák-adjusted), triptorelin treatment in males significantly reduced body mass compared to controls between days 12 and 19 (*p* < 0.05), with an average decrease of 30–35 g (≈10%–12%). In contrast, in females, triptorelin treatment significantly increased body mass from day 18 to 28 (*p* < 0.05), with an average increase of 29–35 g (≈18%–22%).

These findings demonstrate opposite, time-dependent effects of treatment in males and females.

### 3.4. Food Intake and Locomotor Activity

**Figure 4:**
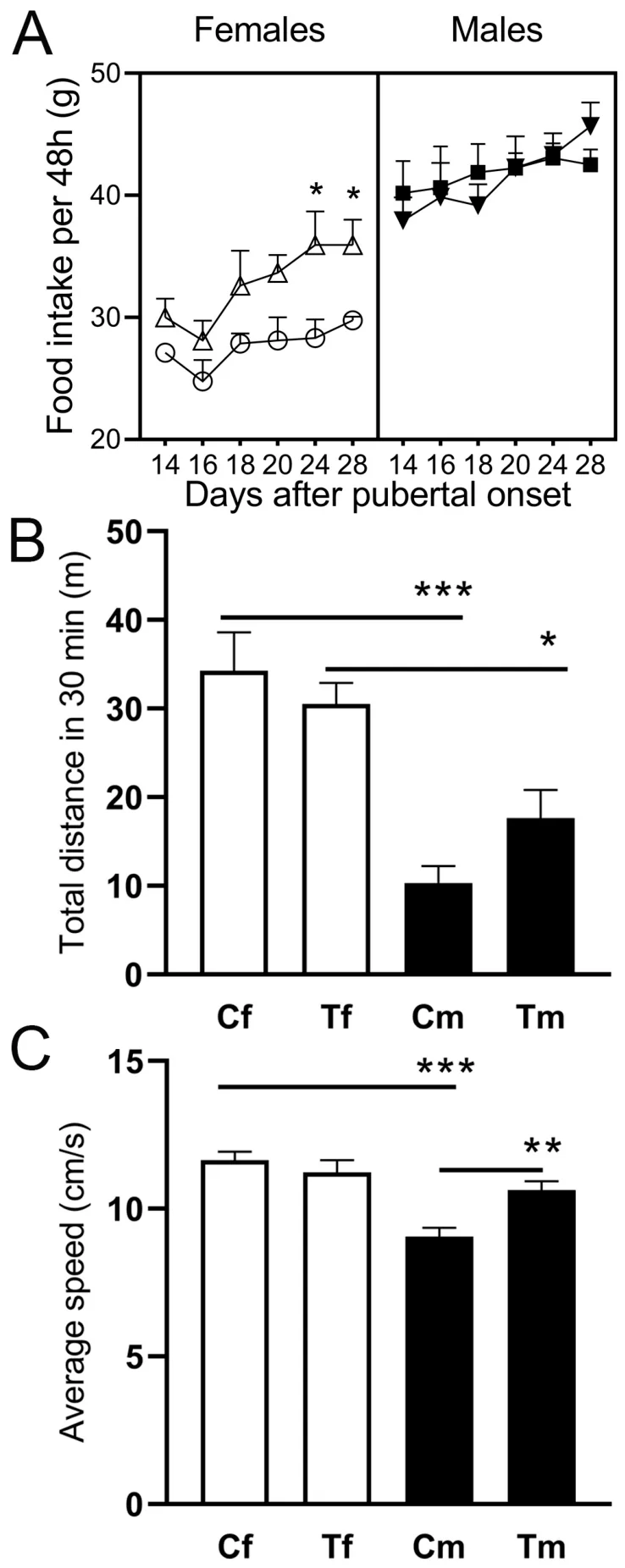
Food intake and locomotor activity under triptorelin treatment. (**A**) Average food intake per cage for female (**left**) and male (**right**) animals. Food intake was significantly higher for the female T group at days 24 and 28. * *p* < 0.05 vs. control at corresponding time points. Open circles, control females; open triangles, treated females; black squares, control males; black triangles, treated males. Food intake in males was not different between the C and T groups. (**B**) Total distance traveled in the open field arena during 30 min for females (open bars) and males (black bars). (**C**) Triptorelin significantly increased average velocity in males but not in females. * *p* < 0.05; ** *p* < 0.01; *** *p* < 0.001. Data are presented as mean ± SEM (*n* = 9 animals (3 cages) per group).

To elucidate the origin of differences in weight gain, we monitored food intake and locomotor activity, as shown in Figure 4. For food intake, a highly significant main effect of sex was observed (F_1,8.02_ = 52.08, *p* < 0.001), with male rats consuming substantially more food than females throughout the study. A significant main effect of day was also observed (F_5,40.02_ = 9.81, *p* < 0.001), reflecting a general increase in food consumption during maturation. The main effect of treatment (F_1,8.02_ = 2.15, *p* = 0.181) and the omnibus three-way interaction (F_5,40.02_ = 0.41, *p* = 0.839) were not significantly different.

However, post hoc pairwise comparisons revealed a sex-specific hyperphagic response that parallels the weight gain trajectory observed in female rats (Figure 4A). In male rats, triptorelin treatment had no significant impact on food consumption at any time point. While food intake did not differ during the early phase on Days 14, 16, and 18 (*p* > 0.05), a statistical trend toward increased food consumption emerged on Day 22 (mean difference = −5.73 g, *p* = 0.055). This hyperphagic effect became statistically significant on Day 24, with treated females consuming significantly more food than controls (mean difference = −7.25 g, *p* = 0.017). Elevated food intake was also recorded on Day 28 (mean difference = −5.92 g, *p* = 0.048).

For locomotor activity, defined as the total distance traveled within the 30 min testing period, a Type III two-way ANOVA showed that the main effect of sex was highly significant (F_1,31_ = 35, *p* < 0.0001; Figure 4B). Conversely, the main effect of triptorelin treatment was not statistically significant (F_1,31_ = 0.34, *p* = 0.57). The sex:treatment interaction showed a statistical trend (F_1,31_ = 3, *p* = 0.08). Similarly, in the analysis of the average speed, the main effect of sex was significant (F_1,31_ = 23, *p* < 0.0001; Figure 4C), whereas the main effect of treatment showed only a statistical trend (F_1,31_ = 3, *p* = 0.09). However, a highly significant sex:treatment interaction effect was observed (F_1,31_ = 9, *p* = 0.005), demonstrating that triptorelin modulates locomotion velocity in a sex-dependent manner.

To resolve specific group differences for both parameters, Tukey post hoc multiple comparisons were conducted. In control groups, robust sexual dimorphism was evident in both tested locomotor measures: control females exhibited higher locomotor activity than control males (predicted LS mean difference = 23.96, *p* < 0.0001) and moved at a significantly faster average speed (predicted LS mean difference = 2.6, *p* < 0.0001).

Triptorelin treatment altered locomotor parameters only in the male cohort: it increased average speed compared to control males (predicted LS mean difference = −1.6, *p* = 0.012), while the corresponding increase in locomotor activity was not statistically significant (predicted LS mean difference = −7.35, *p* = 0.37). In contrast, triptorelin treatment had no impact on females, with control and triptorelin groups showing almost identical levels of both locomotor activity (*p* = 0.82) and average speed (*p* = 0.80).

For total locomotor activity, treated females remained significantly more active than treated males (predicted LS mean difference = 12.87, *p* = 0.03). The triptorelin-induced increase in average speed in males led to the disappearance of the sex difference observed in control groups—treated females no longer differed significantly from treated males (*p* = 0.60), and control females showed no significant difference compared to treated males (*p* = 0.17).

### 3.5. Targeted Gene Expression Profiling of the Hypothalamus

Following the assessment of gonads, pituitary gene expression, and changes in physical activity and energy balance, we focused on the effects of triptorelin on the central regulator of the HPG axis, the hypothalamus.

**Figure 5:**
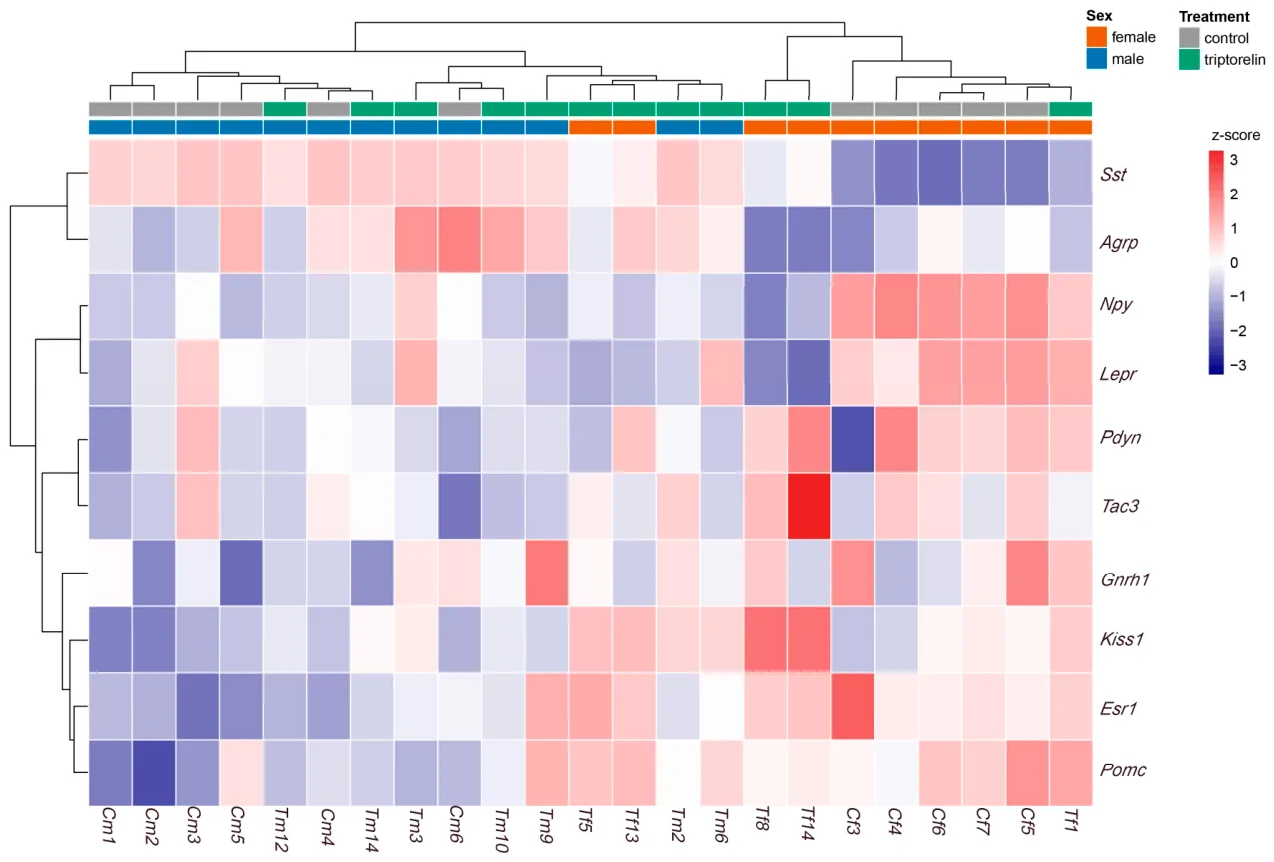
Heatmap of hypothalamic gene expression. Heatmap showing relative expression levels of selected genes (*Sst*, *Agrp*, *Npy*, *Lepr*, *Pdyn*, *Tac3*, *Gnrh1*, *Kiss1*, *Esr1*, and *Pomc*) in hypothalamic samples from control and triptorelin-treated male and female animals. Gene expression values were log_2_-transformed and standardized as z-scores per gene to allow comparison across samples. Rows represent individual genes, while columns represent individual samples (*n* = 23). Samples are annotated by sex (female: orange, male: blue) and treatment (control: gray, triptorelin: green). Hierarchical clustering was performed using Euclidean distance and ward.D2. Color scale: dark blue—downregulation; white—no change; red—upregulation. Experimental group abbreviations: Cf—Female Control; Tf—Female Triptorelin; Cm—Male Control; Tm—Male Triptorelin. Numbers denote individual animals within each group.

To obtain an overview of the global transcriptional landscape across experimental groups, hierarchical clustering was performed on the log_2_-transformed, z-score-normalized expression levels of the 10 target genes (Figure 5).

The samples (horizontal axis on the dendrogram) were primarily divided into two major clusters: (i) the right female cluster contained all control samples (Cf) and the majority of triptorelin-treated females (Tf). This group was characterized by upregulation of *Kiss1*, *Esr1*, and *Pomc* and downregulation of *Sst*; (ii) the left cluster (predominantly males) encompassed all male control samples (Cm) and male triptorelin-treated samples (Tm). Notably, two triptorelin-treated female samples (Tf5 and Tf13) clustered within this group, indicating a treatment-induced shift in these specific animals toward a male-like expression profile.

The 10 target genes were divided into three hierarchical blocks based on their co-expression profiles across sexes and treatments: (i) the *Sst* and *Agrp* cluster—this branch isolates genes involved in energy balance and growth regulation with a sex dimorphic baseline expression pattern, with *Sst* showing higher expression in control males than in control females; (ii) the *Npy* and *Lepr* cluster—this pair shares a downregulation pattern in triptorelin-treated females; (iii) the neuroendocrine cluster—this branch encompasses the remaining six genes, splitting into two sub-groups: the *Pdyn* and *Tac3* sub-cluster, which groups genes exhibiting a moderate main effect of sex; and the *Gnrh1*, *Kiss1*, *Esr1*, and *Pomc* sub-cluster, which represents the primary driving force of sexual dimorphism in the dataset, characterized by higher expression in female controls compared to male controls, together with induction of *Kiss1* in females following triptorelin treatment.

#### 3.5.1. Hypothalamic Genes with Three Significant Effects

*Sst* and *Npy* showed significant main effects for both factors and an interaction effect (Table 4). *Sst* expression was influenced by sex (F_1,19_ = 243.58, *q* < 0.001), treatment (F_1,19_ = 39.85, *q* < 0.001), and a significant sex:treatment interaction (F_1,19_ = 54.65, *q* < 0.001). Triptorelin treatment significantly induced *Sst* expression in females, while it had no significant impact on males (Figure S3 in the Supplementary Materials File). Baseline sexual dimorphism (Cf vs. Cm, *p* < 0.001) remained pronounced following treatment (Tf vs. Tm, *p* < 0.001). *Npy* expression similarly demonstrated significant effects for sex (F_1,19_ = 18.08, *q* = 0.001), treatment (F_1,19_ = 19.45, *q* = 0.001), and their interaction (F_1,19_ = 23.13, *q* = 0.001). According to the post hoc test, the treatment had an effect only in females, causing a significant reduction in *Npy* levels compared to female controls (*p* < 0.001). (Figure S3).

#### 3.5.2. Genes with Two Significant Effects

*Lepr* expression was characterized by a significant main effect of treatment (F_1,19_ = 7.34, *q* = 0.035) and a significant sex:treatment interaction (F_1,19_ = 9.16, *q* = 0.023. Pairwise post hoc comparisons confirmed that triptorelin treatment downregulated *Lepr* expression in females only (*p* = 0.006; Figure S3). *Kiss1* expression was driven independently by sex (F_1,19_ = 28.69, *q* < 0.001) and treatment (F_1,19_ = 40.67, *q* < 0.001), without a significant interaction (Table 4). Post hoc analysis highlighted sexual dimorphism under control conditions (*p* = 0.022). Triptorelin treatment led to an upregulation of *Kiss1* in females (*p* = 0.001) and a similarly significant induction in males (*p* = 0.002; Figure S3).

#### 3.5.3. Genes with One or No Significant Effect

Several genes were modulated by sex only, without responding to triptorelin treatment (Table 4), such as *Esr1* (F_1,19_ = 27.48, *q* < 0.001) and *Pomc* (F_1,19_ = 15.52, *q* = 0.002). Specifically, for *Esr1* we observed a difference between female and male controls (*p* = 0.001), which reached only a statistical trend following treatment (Tf vs. Tm, *p* = 0.052; Figure S3), while for *Pomc*, a baseline difference between sexes was also observed (*p* = 0.009), but no treatment-related modifications were detected (Table 4, Figure S3).

*Pdyn*, *Tac3*, and *Agrp* demonstrated a significant main effect of sex (Table 4). However, the pairwise analyses did not yield statistically significant results (*p* > 0.05 across all comparisons, Figure S3). *Gnrh1* expression did not vary significantly as a function of sex, treatment, or their interaction (Table 4), and post hoc comparisons confirmed the absence of any statistical differences (Figure S3).

## 4. Discussion

We show here that long-acting GnRHa triptorelin effectively suppresses reproductive maturation in both female and male rats. In females, we observed estrous cycle arrest in diestrus, reduced ovarian mass, altered ovarian histology, and a reduced number of antral follicles and CL, findings supported by others [26,27]. Regarding the testicles, triptorelin affected their weight and the diameter of seminiferous tubules, in line with previous studies [28,29,30,31]. In our hands, however, triptorelin partially suppresses spermatogenesis, confirming prolonged and efficient suppression of the HPG axis. The variable degree of spermatogenesis suppression observed in the present study is consistent with the heterogeneous testicular responses previously reported after GnRHa treatment [30]. It is also of note that the effectiveness and physiological consequences of GnRHa treatment partly depend on the developmental stage at treatment initiation. Namely, the HPG axis undergoes rapid maturation during puberty, with changes in GnRH secretion [32], pituitary responsiveness [33], and gonadal sensitivity [34].

Suppression of the HPG axis in both sexes is also evidenced in the pituitary gland of both sexes, as continuous occupancy of the GnRHR produces expected *Gnrhr* and *Lhb* downregulation [35]. On the other hand, continuous GnRH induces *Cga* expression [6], as we observe here. We also anticipated *Fshb* downregulation in both sexes. Interestingly, females do not show *Fshb* mRNA downregulation. However, GnRH is not the only significant regulator of *Fshb*; one possible explanation is a loss of inhibitory tone provided by the ovaries, because prolonged disruption of follicular development may also include a decrease in granulosa cell inhibin production. For example, both immune neutralization of inhibin and ovariectomy lead to an increase in *Fshb* mRNA [36]. It is of note that 28 days after goserelin acetate implantation, mice show FSH blood levels similar to control animals, although the levels were significantly lower on days 9 and 21 [27].

Although circulating gonadotropins and steroid hormones were not measured in the present study, the pronounced reproductive suppression observed here is supported by gonadal morphology and pituitary gene expression. This is consistent with numerous previous studies demonstrating that GnRH agonist treatment suppresses both gonadotropin secretion and gonadal steroid hormones production [37,38,39,40]. Thus, the absence of direct hormone measurements limits the physiological characterization of the response but does not undermine the evidence for reproductive-axis suppression.

The increase in weight gain following GnRHa treatment in females is consistent with previous rodent studies [16,18,41]. Similar sex-specific effects have been reported in humans, where GnRHa treatment transiently increases body mass index in girls, but not boys with CPP [42,43]. These changes are generally reversible and tend to resolve within one year after treatment cessation [43,44,45]. The body mass index increase under GnRHa in girls seems to be linked to weight status prior to treatment [45] and dietary habits [46]. The weight increase has also been suggested to be related to direct effects on human adipocytes through GnRHR [47] or ascribed to a “menopause-effect”, i.e., estrogen deficiency [48]. The here-observed increased body weight gain in triptorelin-treated females may be partly explained by increased food intake, although the contribution of altered energy expenditure cannot be excluded.

The altered feeding behavior of triptorelin-treated females was accompanied by decreased hypothalamic *Lepr* expression, suggesting reduced hypothalamic leptin responsiveness. This could be attributed to estradiol deficiency, similar to findings from rat ovariectomy models [49,50], and also correlates with increased leptin levels in girls with CPP under GnRHa treatment [51,52]. We expected to associate changes in *Lepr* expression with alterations in the mRNA expression of orexigenic and anorexigenic peptides in the hypothalamus or *Tshb* in the pituitary gland. However, *Pomc* and *Agrp* remain unchanged, while *Npy* mRNA in females shows paradoxical downregulation under triptorelin treatment. Although reduced leptin signaling is typically associated with increased hypothalamic *Npy* expression during negative energy balance, the concomitant decrease in both *Lepr* and *Npy* expression observed here does not resemble a classical fasting-induced transcriptional response. Interestingly, a recent study finds that in girls with CPP, leuprorelin lowers NPY serum levels but increases circulating leptin and body fat percentage [52]. The increased hypothalamic *Sst* expression observed here is consistent with a previous report demonstrating increased GH pulsatility in females following triptorelin treatment [53], which may enhance GH-mediated negative feedback on hypothalamic somatostatin neurons [54]. However, because somatostatin is expressed by several hypothalamic neuronal populations with distinct roles in energy homeostasis [55], increased *Sst* expression may also contribute, at least in part, to the hyperphagic response observed in females.

As Guarraci et al. [19] already reported, we also observe elevated *Kiss1* mRNA levels in the hypothalamus of the triptorelin-treated animals. This upregulation is not accompanied by changes in the expression of *Pdyn* and *Tac3*, which code for prodynorphin and neurokinin B, stored within kisspeptin neurons in the arcuate nucleus. Kisspeptin neurons in the arcuate nucleus not only regulate reproduction but also participate in circuits governing feeding behavior and energy expenditure. Kisspeptin effects on feeding depend on developmental stage and hormonal status—namely, the kisspeptin-KISS1R system matures during puberty [56]. Even though the mode of action of kisspeptin is mostly anorexigenic, mediated through inhibition of NPY and stimulation of POMC neuron firing [57], our findings suggest that disruption of the reproductive axis during this developmental window may be associated with sex-specific adaptations in hypothalamic pathways involved in feeding regulation. An important limitation of the present study is that gene expression was analyzed in whole hypothalamic tissue rather than in individual hypothalamic nuclei. Consequently, nucleus-specific transcriptional changes may have been underestimated or masked by opposing responses in anatomically distinct neuronal populations. Furthermore, our hypothalamic analyses are based on mRNA expression without corresponding protein or functional validation. Further studies are currently underway to determine whether the observed transcriptional changes are accompanied by alterations at the protein and functional levels.

Unlike females, male rats exhibited no evidence of coordinated transcriptional remodeling of the hypothalamic pathways examined, suggesting that the lag in body weight gain may involve mechanisms other than altered hypothalamic expression of these metabolic regulators. Similarly, prepubertal male rats gained less weight after leuprorelin administration [18]. Nevertheless, we find that this effect is transient, and indeed, longer triptorelin administration seems to result in increased weight gain in male rats as well [53]. It should be noted that the present study evaluated a relatively short exposure period of 28 days. Ongoing studies are addressing the longer-term consequences of pubertal triptorelin exposure and whether the observed neuroendocrine alterations persist or normalize following treatment.

In the present study, rats display a distinct sex-specific behavioral pattern characterized by increased locomotor activity in females compared to males, largely in accordance with literature data [58,59]. Our findings suggest subtle alterations in exploratory behavior rather than a robust increase in overall locomotor activity. Triptorelin induced higher average speed in males; however, the increase in total distance traveled showed only a trend. Whereas increased ambulation has been reported in mice following two weeks of leuprorelin treatment [15], locomotor behavior in our study was evaluated at a later time point after triptorelin administration. It is therefore possible that the behavioral consequences of pubertal GnRHa treatment evolve over time, although differences in species, experimental protocols, and GnRHa formulations may also contribute to the observed discrepancies. While the mechanisms underlying these subtle behavioral outcomes following triptorelin treatment remain to be elucidated, the present findings suggest that suppression of the HPG axis may underlie the altered locomotion patterns, possibly through alterations in energy expenditure and basal metabolism, as reflected in transiently reduced body weight in male rats. Previously, triptorelin treatment has been reported to reduce voluntary wheel-running activity in young rats [60], a behavior that reflects not only motor output but also motivation to engage in rewarding physical activity. These findings imply that GnRHa may differentially influence neural circuits regulating exploratory behavior and voluntary exercise. This interpretation is further supported by evidence that peripubertal but not adult castration increases locomotor behavior in adulthood [61], indicating an organizational role of pubertal testosterone in shaping adult activity patterns. Notably, testosterone deficiency induced by castration during adulthood is not associated with changes in locomotor activity [62], suggesting that early-life suppression of gonadal hormones has distinct consequences due to disruption of pubertal organizational processes, further supporting the importance of carefully interpreting the findings of hormonal manipulation during puberty.

Collectively, these findings indicate that the consequences of GnRHa treatment during puberty extend beyond suppression of reproductive function and are accompanied by sex-specific changes in hypothalamic gene expression related to metabolic regulation. However, because the present study cannot distinguish direct effects of triptorelin from secondary effects of gonadal steroid suppression, the basis of these transcriptional changes remains unclear. Further studies incorporating hormone replacement and functional analyses will be needed to determine the mechanisms underlying these observations.

## 5. Conclusions

In conclusion, triptorelin effectively suppresses pubertal maturation in both sexes but elicits fundamentally different metabolic adaptations in males and females. Female rats develop hyperphagia, increased body weight, and coordinated changes in hypothalamic *Lepr*, *Npy*, and *Sst* expression, whereas males exhibit a transient reduction in body weight gain and subtle behavioral alterations without comparable changes in the expression of the hypothalamic genes examined. These findings suggest that the metabolic consequences of pubertal GnRHa treatment extend beyond reproductive suppression and involve sex-specific reorganization of hypothalamic circuits regulating energy homeostasis. Further studies examining individual hypothalamic nuclei and endocrine pathways, particularly the GH/IGF-1 axis and leptin signaling, will be essential for defining the mechanisms underlying these developmental effects.

## Figures and Tables

**Table 1:** Primer sequences for q-PCR.

Name	Coding for	Accession Number	Primer Sequences
*Gapdh*	glyceraldehyde 3-phosphate dehydrogenase	NM_017008.4	f: CAACTCCCTCAAGATTGTCAGCAA r: GGCATGGACTGTGGTCATGA
*Gnrh1*	gonadotropin-releasing hormone	NM_012767.2	f: AGGAGGATCAAATGGCAGAAC r: TCTTCAATCAGACGTTCCAGAGC
*Kiss1*	kisspeptin	NM_181692.2	f: TCCTCTGTGTGGCCTCTTTT r: AGGCTTGCTCTCTGCATACC
*Npy*	neuropeptide Y	NM_012614.2	f: TACTACTCCGCTCTGCGACA r: GGGCATTTTCTGTGCTTTCT
*Sst*	somatostatin	NM_012659.2	f: GATAGCGGCTGAAGGAGACG r: CAAAGCCAGGACGATGCAGA
*Lepr*	leptin receptor	NM_001429462.1	f: GGGACATAGAGTGCTGGATGA r: GGTGAACCTTAGAGTCATAATTCTTG
*Agrp*	agouti-related peptide	NM_033650.1	f: TGTGTAAGGCTGCACGAGTC r: AGTACCTAGCTTGCGGCAGT
*Pomc*	proopiomelanocortin	NM_139326.3	f: AGAACGCCATCATCAAGAACG r: AGGTCAGGTGCTCTCGCC
*Tshb*	thyroid stimulating hormone beta subunit	NM_013116.2	f: ACAGAACGGTGGAAATACCG r: GTTGGTTTTGACAGCCTCGT
*Esr1*	estrogen receptor alpha	NM_012689.2	f: GCGCAAGTGTTACGAAGTGG r: AGTGCCCATTTCATTTCGGC

**Table 2:** Taqman probes for q-PCR.

Name	Coding for	Probe Identification Number
*Gapdh*	glyceraldehyde 3-phosphate dehydrogenase	Rn01462661_g1
*Cga*	glycoprotein hormones alpha subunit	Rn01440184_m1
*Lhb*	luteinizing hormone beta subunit	Rn00563443_g1
*Fshb*	follicle stimulating hormone beta subunit	Rn01484594_m1
*Gnrhr*	gonadotropin-releasing hormone receptor	Rn00563377_m1
*Prl*	prolactin	Rn00561791_m1
*Pdyn*	prodynorphin	Rn00571351_m1
*Tac3*	neurokinin B	Rn00569758_m1

**Table 3:** Statistical summary of two-way ANOVA for pituitary gene expression.

Gene	Factor	Sum of Squares	df (Num, Den)	F-Value	*q*-Value
*Lhb*	**sex**	2.77	1, 21	9.76	**0.009**
	**treatment**	15.74	1, 21	55.41	**<0.001**
	sex:treatment	1.17	1, 21	4.12	0.130
*Fshb*	**sex**	19.71	1, 21	32.61	**<0.001**
	**treatment**	7.70	1, 21	12.75	**0.003**
	sex:treatment	3.80	1, 21	6.28	0.072
*Gnrhr*	sex	0.29	1, 21	1.85	0.220
	**treatment**	13.22	1, 21	85.49	**<0.001**
	sex:treatment	0.14	1, 21	0.91	0.409
*Cga*	**sex**	2.65	1, 21	25.15	**<0.001**
	**treatment**	7.40	1, 21	70.21	**<0.001**
	sex:treatment	0.87	1, 21	8.22	0.065
*Tshb*	sex	0.02	1, 21	0.11	0.745
	treatment	0.42	1, 21	2.79	0.128
	sex:treatment	0.002	1, 21	0.02	0.905
*Pomc*	**sex**	1.84	1, 21	6.93	**0.022**
	treatment	0.05	1, 21	0.17	0.686
	sex:treatment	0.25	1, 21	0.96	0.409
*Prl*	**sex**	30.99	1, 21	33.20	**<0.001**
	**treatment**	5.57	1, 21	5.96	**0.033**
	sex:treatment	3.23	1, 21	3.46	0.134

The table shows the results of a two-way ANOVA (Type III Sum of Squares) assessing the effects of sex, triptorelin treatment, and their interaction on the mRNA expression of target genes. For each gene, the Sum of Squares, degrees of freedom (df), numerator, denominator (Num, Den), F-statistics (F-value), and Benjamini–Hochberg adjusted *p*-values (*q*-values) are reported. Statistical significance was defined as *q* < 0.05 and indicated in bold.

**Table 4:** Statistical summary of two-way ANOVA for gene expression in the hypothalamus.

Gene	Factor	Sum of Squares	df (Num, Den)	F-Value	*q*-Value
*Sst*	**sex**	37.08	1, 19	243.58	**<0.001**
	**treatment**	6.07	1, 19	39.85	**<0.001**
	**sex:treatment**	8.32	1, 19	54.65	**<0.001**
*Npy*	**sex**	2.73	1, 19	18.08	**0.001**
	**treatment**	2.94	1, 19	19.45	**0.001**
	**sex:treatment**	3.50	1, 19	23.13	**0.001**
*Lepr*	sex	0.07	1, 19	0.39	0.542
	**treatment**	1.32	1, 19	7.34	**0.035**
	**sex:treatment**	1.65	1, 19	9.16	**0.023**
*Kiss1*	**sex**	10.20	1, 19	28.69	**<0.001**
	**treatment**	14.46	1, 19	40.67	**<0.001**
	sex:treatment	0.20	1, 19	0.57	0.576
*Pdyn*	**sex**	0.77	1, 18	5.32	**0.044**
	treatment	0.02	1, 18	0.14	0.791
	sex:treatment	0.02	1, 18	0.11	0.748
*Tac3*	**sex**	0.26	1, 19	5.15	**0.044**
	treatment	0.04	1, 19	0.88	0.514
	sex:treatment	0.02	1, 19	0.31	0.649
*Esr1*	**sex**	1.59	1, 19	27.48	**<0.001**
	treatment	0.23	1, 19	3.89	0.126
	sex:treatment	0.11	1, 19	1.84	0.386
*Pomc*	**sex**	2.05	1, 19	15.52	**0.002**
	treatment	0.32	1, 19	2.43	0.226
	sex:treatment	0.20	1, 19	1.53	0.386
*Agrp*	**sex**	1.31	1, 18	6.61	**0.032**
	treatment	0.004	1, 18	0.02	0.885
	sex:treatment	0.12	1, 18	0.62	0.576
*Gnrh1*	sex	0.29	1, 19	1.78	0.220
	treatment	0.03	1, 19	0.16	0.791
	sex:treatment	0.26	1, 19	1.61	0.386

The table shows the results of a two-way ANOVA (Type III Sum of Squares) assessing the effects of sex, triptorelin treatment, and their interaction on the mRNA expression of target genes in the hypothalamus. For each gene, the Sum of Squares, degrees of freedom (df), numerator, denominator (Num, Den), F-statistics (F-value), and Benjamini–Hochberg adjusted *p*-values (*q*-values) are reported. Statistical significance was defined as *q* < 0.05 and indicated in bold.

## Data Availability

The raw data supporting the conclusions of this article will be made available by the authors on request. The datasets are not publicly available because they contain unpublished data that form part of ongoing research and future planned analyses.

## Supplementary Materials

The following supporting information can be downloaded at: https://www.mdpi.com/article/10.3390/biology15161376/s1, Figure S1: Triptorelin depot induced changes in gonads in both sexes after 28 days; Figure S2: Box plots of gene expression profile in the pituitary, shown in the heatmap in Figure 2; Figure S3: Box plots of gene expression profile in the hypothalamus, shown in the heatmap in Figure 5.

## Abbreviations

The following abbreviations are used in this manuscript:

AgRP	Agouti-Related Peptide
CL	Corpus Luteum
CPP	Central Precocious Puberty
FSH	Follicle-Stimulating Hormone
GH	Growth Hormone
GnRH	Gonadotropin-Releasing Hormone
GnRHa	Gonadotropin-Releasing Hormone Agonist
GnRHR	Gonadotropin-Releasing Hormone Receptor
HPG	Hypothalamic–Pituitary–Gonadal (axis)
IGF-1	Insulin-like Growth Factor 1
LH	Luteinizing Hormone
NPY	Neuropeptide Y
PND	Postnatal Day
POMC	Proopiomelanocortin
