# Supplementary material for: Pubertal Triptorelin Exposure Alters Hypothalamic Reproductive–Metabolic Integration in a Sex-Specific Manner
Source: Biology (Basel). 2026 Aug 12;15(16):1376. doi: 10.3390/biology15161376 (PMC13509935; doi:10.3390/biology15161376)
Supplement: Supplementary file 1 [file biology-15-01376-s001.zip › biology-4475356-supplementary.pdf]

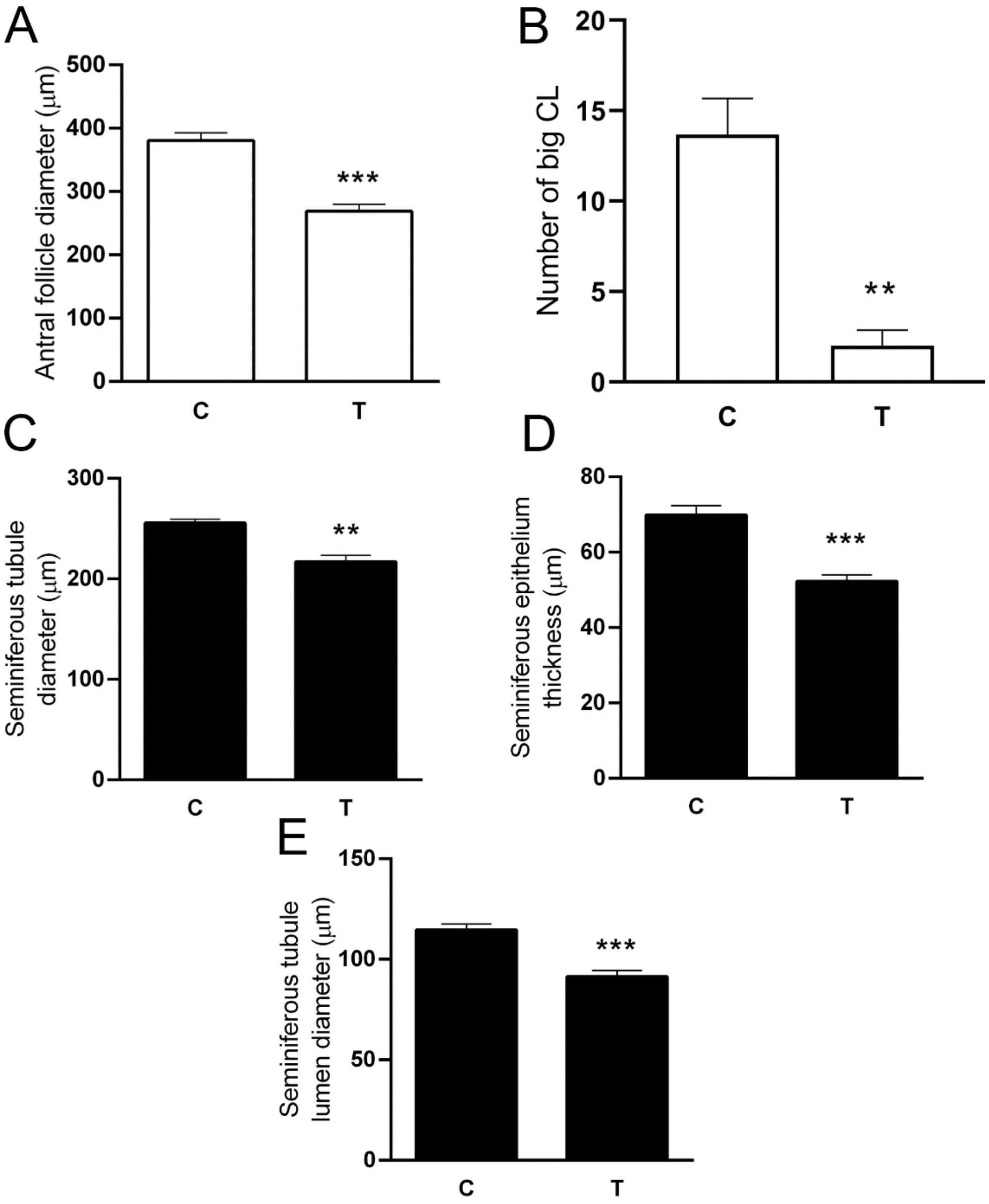

Figure S1. Triptorelin depot induced changes in gonads in both sexes after 28 days. (**A**) Mean diameter of antral follicles in control (C) and triptorelin treated (T) animals. (**B**) Number of corpora lutea over 1000 μm in diameter. (**C**) Mean diameter of seminiferous tubules in control (C) and triptorelin treated (T) animals. (**D**) Mean seminiferous epithelium thickness in C and T groups. (**E**) Mean diameter of seminiferous tubule lumen in C and T groups. Results presented as mean ± SEM, $n \geq 6$ /group. ** $p < 0.01$, *** $p < 0.005$; Welch's t-test.

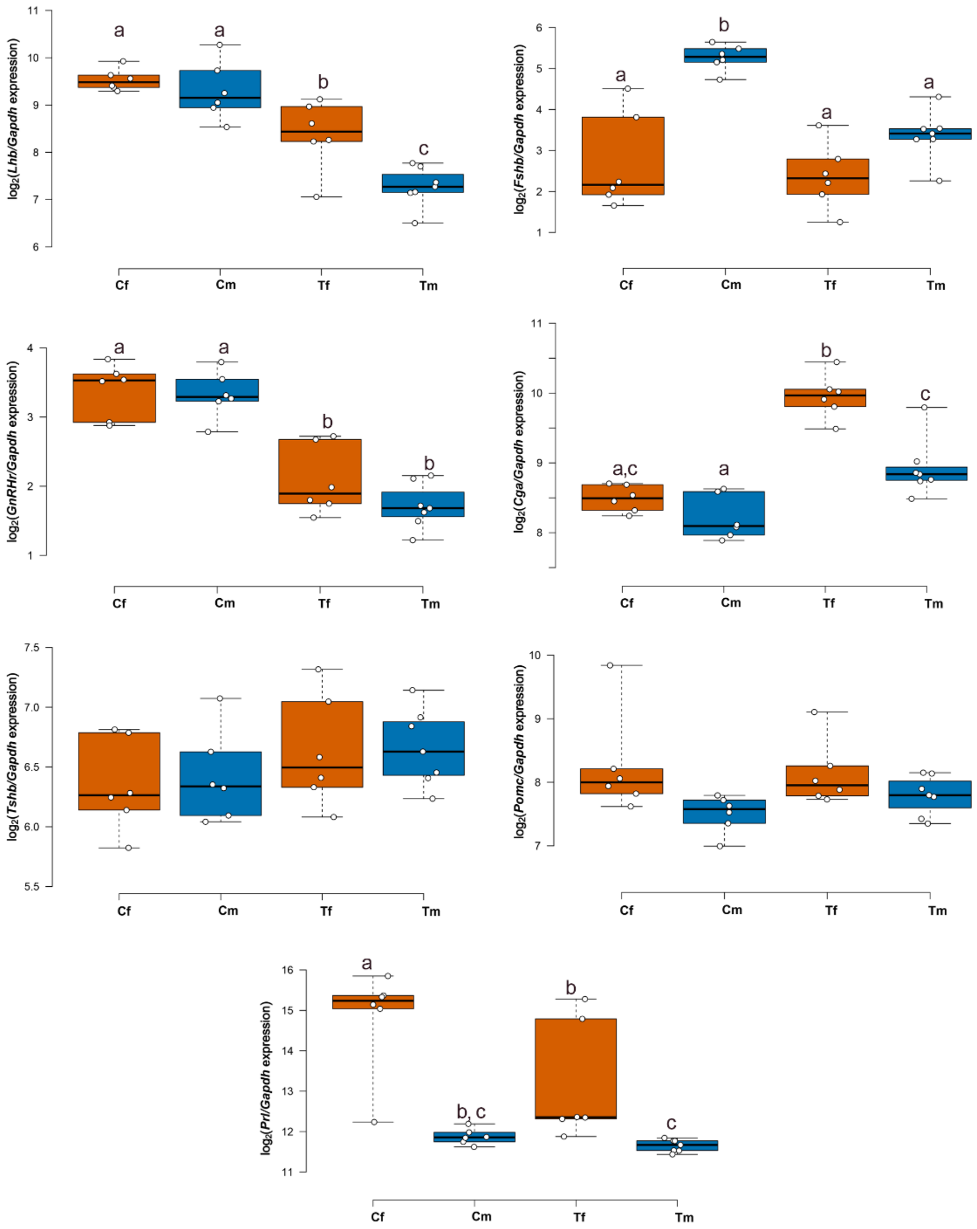

Figure S2. Box plots of gene expression profile in the pituitary, shown in the heatmap in Figure 2. The results are presented as log_2-transformed expression of target genes relative to *Gapdh*. Different letters above whiskers indicate statistically significant differences between groups according to Tukey's post hoc test following the Type III two-way ANOVA. Experimental group abbreviations: Cf − Female Control; Tf − Female Triptorelin; Cm − Male Control; Tm − Male Triptorelin.

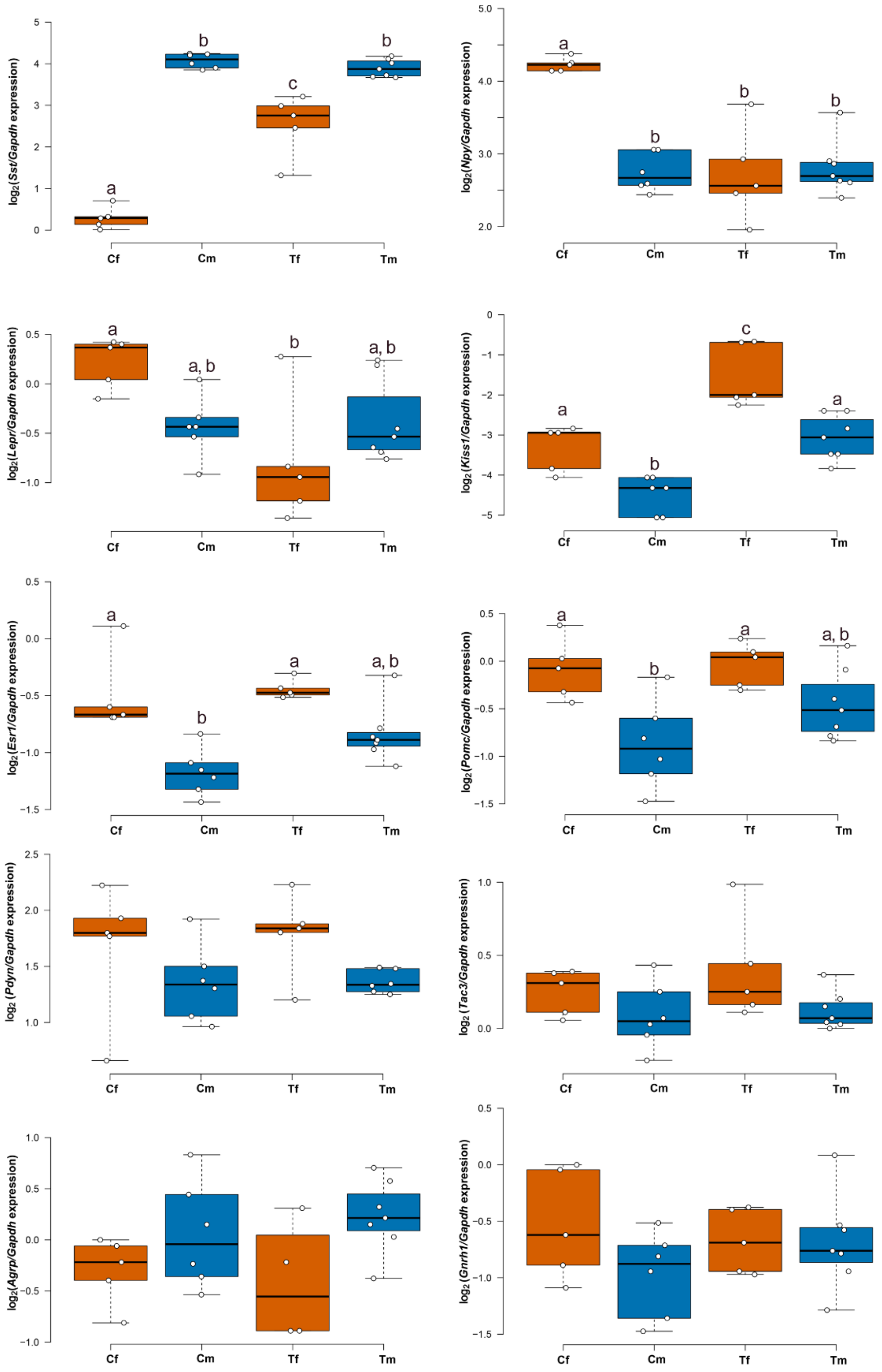

Figure S3. Box plots of gene expression profile in the hypothalamus, shown in the heatmap in Figure 5. The results are presented as $\log_2$-transformed expression of target genes relative to *Gapdh*. Different letters above whiskers indicate statistically significant differences between groups according to Tukey's post hoc test following the Type III two-way ANOVA. Experimental group abbreviations: Cf − Female Control; Tf − Female Triptorelin; Cm − Male Control; Tm − Male Triptorelin.
